# Synthesis and Sintering Reaction Mechanism of High-Performance MgO-CaO-Fe_2_O_3_ Clinker

**DOI:** 10.3390/ma16052086

**Published:** 2023-03-03

**Authors:** Yunqin Gao, Jiawei Wang, Xiaoli Tian, Yanlong Yang, Xing Hou

**Affiliations:** 1 College of Materials Science and Engineering, Xi’an University of Architecture & Technology, Xi’an 710055, China; 2 Puyang Punai High-Temperature Materials Co., Ltd., Puyang 457000, China

**Keywords:** magnesite, MgO-CaO-Fe_2_O_3_ clinker, microstructure, HSC chemistry 6, synthesis mechanism

## Abstract

High-performance MgO-CaO-Fe_2_O_3_ clinker was prepared using magnesite from Xinjiang (with high calcium and low silica), calcium oxide, and ferric oxide as raw materials. Microstructural analysis and thermogravimetric analysis, combined with HSC chemistry 6 software simulations, were used to investigate the synthesis mechanism of MgO-CaO-Fe_2_O_3_ clinker and the effect of firing temperature on the properties of MgO-CaO-Fe_2_O_3_ clinker. The results show that MgO-CaO-Fe_2_O_3_ clinker with a bulk density of 3.42 g·cm^−3^, water absorption of 0.7%, and excellent physical properties can be formed by firing at 1600 °C for 3 h. In addition, the crushed and reformed specimens can be refired at temperatures of 1300 °C and 1600 °C to achieve compressive strengths of 17.9 MPa and 39.1 MPa, respectively. The main crystalline phase of the MgO-CaO-Fe_2_O_3_ clinker is the MgO phase; the 2CaO·Fe_2_O_3_ phase generated by the reaction is distributed between the MgO grains to form a cemented structure with a small quantity of 3CaO·SiO_2_ and 4CaO·Al_2_O_3_·Fe_2_O_3_ also distributed between the MgO grains. A series of decomposition and resynthesis chemical reactions occurred during the firing of the MgO-CaO-Fe_2_O_3_ clinker, and the liquid phase appeared in the system once the firing temperature exceeded 1250 °C. The presence of the liquid phase promoted intergranular mass transfer between the MgO grains, ensuring the continuous growth of the MgO grains and furthering the densification of the MgO-CaO-Fe_2_O_3_ clinker.

## 1. Introduction

MgO-CaO-Fe_2_O_3_ clinker ((MCF)cl), as one of the raw materials for the preparation of the MgO-CaO-Fe_2_O_3_ system ramming mix for the bottom of the electric arc furnace, has a significant impact on the service life and safe production of the electric arc furnace. Due to its direct contact with steel and slag, the refractory at the bottom and slope of the electric arc furnace is subjected to chemical corrosion and scouring by the melt, the impact of the charge, and to rapid cooling and heating. This harsh service environment requires refractories with a good resistance to corrosion and mechanical and thermal stresses, as well as a good sintering ability, high density after firing, and good volumetric stability. The MgO-CaO-Fe_2_O_3_ ramming mix performs excellently and thus matches the requirements of refractories for electric arc furnaces; it is currently the most widely used refractory material for furnace bottoms and slopes [1,2,3,4].

The main minerals composing (MCF)cl are the periclase phase (MgO) and the dicalcium ferrate phase (2CaO·Fe_2_O_3_). The 2CaO·Fe_2_O_3_ is often melted at low temperatures in an oxidizing atmosphere (a minimum temperature of 1250 °C is required for the formation of the liquid phase [5,6]) when the construction of the electric arc furnace bottom pounding material is completed for initial use, or in the early stages of melting per furnace, making the working surface of the furnace bottom pounding material sinter rapidly to form a ceramic bond. In contrast, 2CaO·Fe_2_O_3_ decomposes and melts in a reducing atmosphere during electric furnace melting operations [7]:

$$2CaO\cdot Fe_{2}O_{3}\to CaO+Liquid$$

The FeO that enters the molten-liquid phase after decomposition is absorbed by the MgO, and two high-temperature solid phases of (Mg·Fe)O solid solution and CaO are generated at the working surface of the pounding material. Meanwhile, the crystal size of the MgO grains, and the degree of direct bonding between them, increase due to the absorption of FeO, indicating that the pounding material has good high-temperature sintering and serviceability [8].

The initial (MCF)cl was produced in Europe from raw materials sourced from the Alps (CaO 30%, Fe_2_O_3_ 7%), and then transformed by Liao Magnesium Company to support the distribution of mineral resources in China, through the development and domestic production of a synthetic clinker containing 7~9% CaO and 5~7% Fe_2_O_3_ [9]. Both products are used with a suitable quantity of high-purity magnesium clinker to make a dry MgO-CaO-Fe_2_O_3_ pounding material. Due to the different contents of CaO and Fe_2_O_3_ in the products, they are often used in different furnace conditions. In recent years researchers have focused their attention on comparing the performance and optimization of the use of these two materials for refractories used on electric furnace bottoms [10,11,12,13]. The performance, preparation, and sintering mechanism of (MCF)cl, which directly determines the quality of ramming mixes, have been less studied.

During the preparation of (MCF)cl, a series of chemical reactions take place between the raw materials. Whether and how these reactions take place, and in what order they occur, will determine the course of the sintering reaction of the synthetic clinker. Thermodynamic models combined with thermodynamic databases can reliably predict the course of reactions and the phase composition of products; they are widely used in the fields of materials, metallurgy, chemicals, geology, and environmental engineering. Lothenbach [14] et al. summarized how the Cemdata18 database can be used to reliably calculate the type, composition, quantity, and volume of hydrates formed during the hydration and degradation of the gelling system. Matschei et al. [15] studied the role of calcium carbonate in cement hydration by combining thermodynamic calculations with experimental data. Glasser et al. [16] developed a thermodynamic model to study the effects of composition and temperature on hydrate and pore solutions. Wang et al. [17] systematically investigated the effect of different temperatures, pressures, and slag components on the slag alkali capacity using thermodynamic calculations. These studies all demonstrate the effective application of thermodynamic analyses.

For this paper, high-performance (MCF)cl was prepared via a one-step sintering method using magnesite with high calcium and low silica from Xinjiang, calcium oxide, and ferric oxide as the raw materials. The synthesis mechanism of (MCF)cl and the effect of firing temperature on the properties of the prepared (MCF)cl were investigated using microstructure analysis and TG-DSC, combined with HSC chemistry 6 thermodynamic simulations, with the intention of providing theoretical guidance for the practical production of (MCF)cl.

## 2. Experiment

### 2.1. Preparation of MgO-CaO-Fe_2_O_3_ Clinker

**Figure 1:**
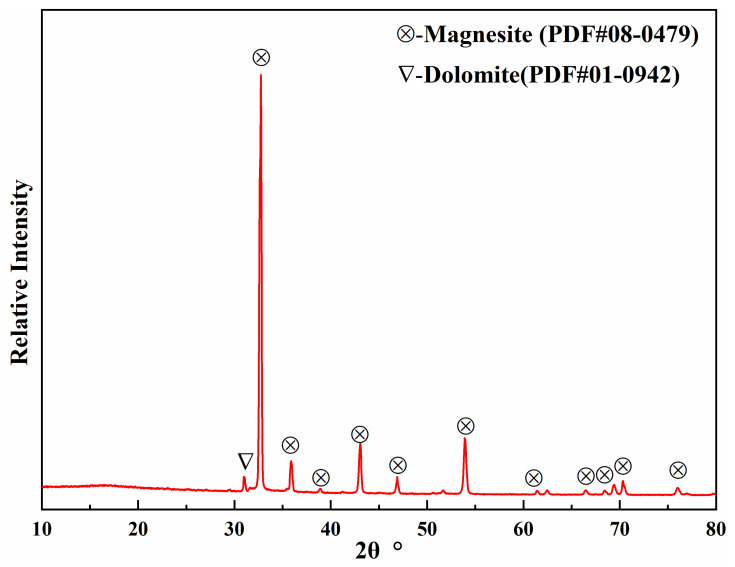
Diffraction results for the magnesite.

Magnesite with high calcium and low silica from Xinjiang, calcium oxide (CaO), and ferric oxide (Fe_2_O_3_) were used as raw materials for the preparation of high-performance (MCF)cl. Table 1 and Figure 1 show the results of the chemical composition and phase analysis of the magnesite, respectively. The magnesite was crushed, finely ground, and sieved before the preparation, and a fine powder (≤0.074 mm) was taken for preparation after sieving. The calcium oxide was digested and aged for 24 h with approximately 50 wt% water. The specimens were prepared according to the DHL-81 grade in the standard “MgO-CaO-Fe_2_O_3_ System Synthetic Material for Electric Furnace Bottom (YB/T 101-2018)”. The fine powdered magnesite, digested CaO, and Fe_2_O_3_ were mixed and ball milled for 4 h. The binding agent was then added and mixed well. An aqueous solution of calcium lignosulfonate was used as the binder, with a density of 1.20 g·cm^−3^. The raw material was pressed under a hydraulic pressure tester at 100 MPa to obtain a billet of Φ 36 × 36 mm. The billets were dried at 110 °C for 24 h and then fired in a high-temperature furnace. The firing temperatures were 1500 °C, 1550 °C, 1600 °C, and 1650 °C. Once they reached the firing temperature, the billets were held there for 3 h before being cooled to room temperature, while remaining in the furnace.

The (MCF)cl obtained by firing was crushed to obtain different particle sizes, such as ≤0.074 mm, 0–1 mm, 1–3 mm, and 3–5 mm, referring to the requirements of the standard (YB/T 101-2018). The different particle sizes of (MCF)cl were taken and mixed proportionally and were then formed into Φ 36 × 36 mm specimens under 100 MPa pressure. After the drying process described above, the specimens were fired in a high-temperature furnace at 1300 °C and 1600 °C for 3 h to obtain refired specimens.

### 2.2. Characterization of the Prepared Specimens

(MCF)cl with a particle size of 3–5 mm was taken to determine the bulk density and water absorption according to the standard “Test method for bulk density of refractory particles (GB/T 2997-2000)”. Using the refired (MCF)cl specimens, the heating-permanent line variation and the compressive strength were measured according to standards “Test method for refired line variation of dense and shaped refractory products (GB/T 5988-2008)” and “Test method for room temperature compressive strength of dense and shaped refractory products, part 2 Liner test method (GB/T 5072.2-2008)”, respectively. An X-ray diffractometer (XRD, model D/MAX-2400) was used for phase characterization of the specimens under the following test conditions: Cu targets Kα radiation at a wavelength of 0.154 nm and a scan rate of 0.02°/s. A field-emission scanning electron microscope (SEM, SU6600) equipped with an X-ray energy spectrometer (EDS) was used to observe the microstructure of the specimens and for compositional analysis. A comprehensive thermal analysis of magnesite and (MCF)cl raw materials was carried out using a comprehensive thermal analyzer (NETZSCHSTA-449F3) with a nitrogen atmosphere, a heating rate of 5 °C/min, and a particle size of ≤0.074 mm.

## 3. Results and Discussion

### 3.1. Performance of the Prepared MgO-CaO-Fe_2_O_3_ Clinker

**Figure 2:**
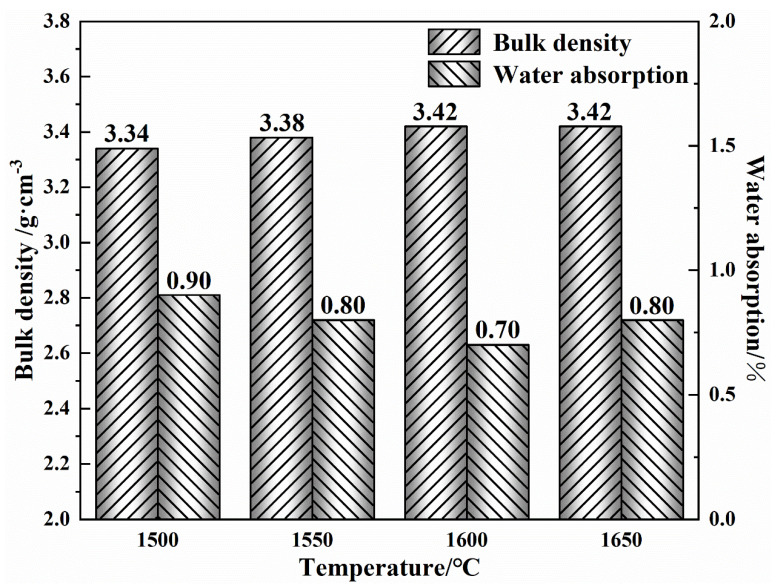
Bulk density and water absorption of the MgO-CaO-Fe_2_O_3_ clinker fired at different temperatures.

**Figure 3:**
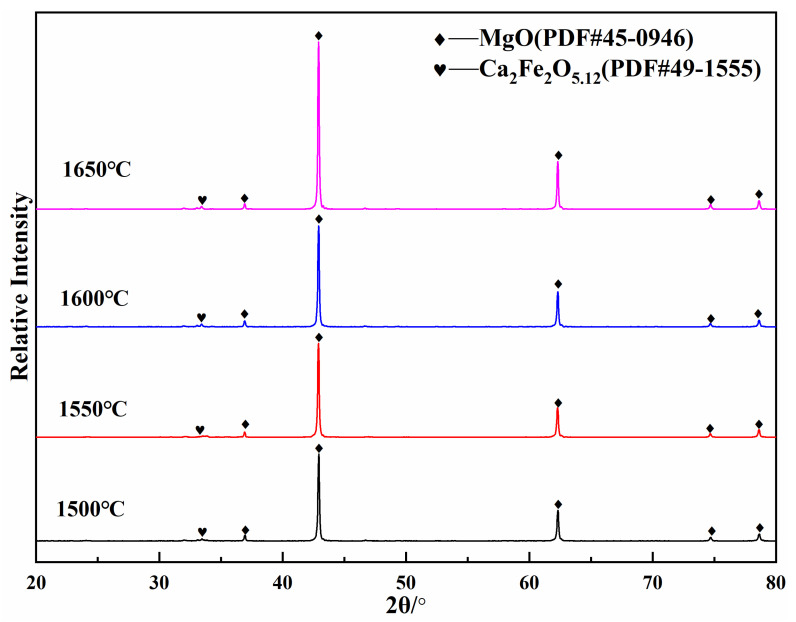
Diffraction results for the MgO-CaO-Fe_2_O_3_ clinker fired at different temperatures.

Figure 2 shows the bulk density and water absorption of (MCF)cl after firing at different temperatures. As can be seen from the figure, the bulk density of (MCF)cl particles gradually increases as the firing temperature rises, reaching a maximum value of 3.42 g·cm^−3^ at 1600 °C. As the firing temperature continues to rise to 1650 °C, the bulk density of the (MCF)cl no longer changes. At this point, the water absorption of (MCF)cl first decreases and then increases with the rise of firing temperature, and reaches a minimum value of 0.7% at 1600 °C. From this, it is clear that the (MCF)cl prepared by magnesite with high calcium and low silica from Xinjiang, calcium oxide, and ferric oxide has a high density and low water absorption when the firing temperature is 1600 °C. Figure 3 shows the diffraction analysis results for (MCF)cl prepared at different firing temperatures. The results show that only the periclase phase (MgO) and the dicalcium ferrate phase (2CaO·Fe_2_O_3_) can be found after firing, and that the phases of fired (MCF)cl do not change significantly with the increasing firing temperature. This indicates that the ingredients have reacted fully during the firing process at high temperatures. (MCF)cl has strict limits on the quantity of SiO_2_ and Al_2_O_3_, according to the environment in which it is used. A large quantity of SiO_2_ will react with CaO to form 2CaO·SiO_2_ and 3CaO·SiO_2_, which inhibit the formation of the cemented phase 2CaO·Fe_2_O_3_ and thus impair the properties of (MCF)cl. Meanwhile, the presence of Al_2_O_3_ reacts with CaO and Fe_2_O_3_ to form the low melting point 4CaO·Al_2_O_3_·Fe_2_O_3_, which as a stable high-temperature liquid phase can significantly affect the corrosion resistance of the material [5]. Due to the extremely low contents of Al_2_O_3_ and SiO_2_ in the magnesite used in this experiment, as shown in Table 1, the phases 3CaO·SiO_2_, 2CaO·SiO_2_ and 4CaO·Al_2_O_3_·Fe_2_O_3_ did not appear in the diffraction phase analysis results after firing.

**Figure 4:**
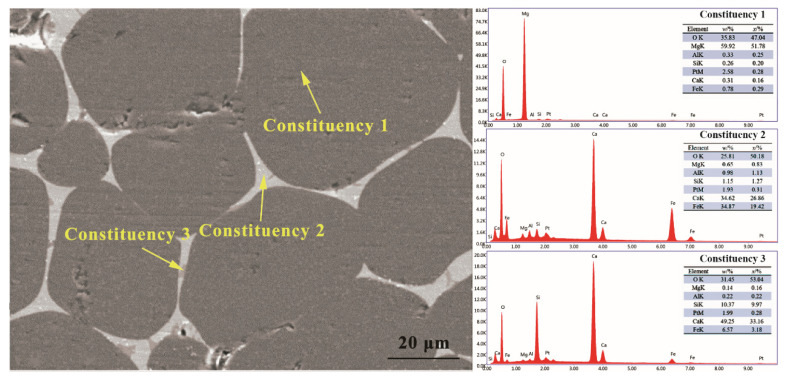
Microstructure and energy spectrum analysis of the MgO-CaO-Fe_2_O_3_ clinker.

Figure 4 shows the microstructure and energy spectrum analysis of (MCF)cl after firing at 1600 °C. As can be seen in the figure, the fired specimens appear in three different colored areas: a dark grey area (constituency 1), a white area (constituency 2), and a light gray area (constituency 3). By combining the results of the energy spectrum analysis with the relative atomic ratios of the phases of MgO, 2CaO·Fe_2_O_3_, 3CaO·SiO_2_, and 4CaO·Al_2_O_3_·Fe_2_O_3_ in (MCF)cl, we can determine that the dark gray area represents MgO with traces of Fe_2_O_3_ in solid solution, the white area is mainly 2CaO·Fe_2_O_3_ with small quantities of 3CaO·SiO_2_, 4CaO·Al_2_O_3_·Fe_2_O_3_, and MgO, and the light gray area is 3CaO·SiO_2_ with small quantities of 2CaO·Fe_2_O_3_.

**Figure 5:**
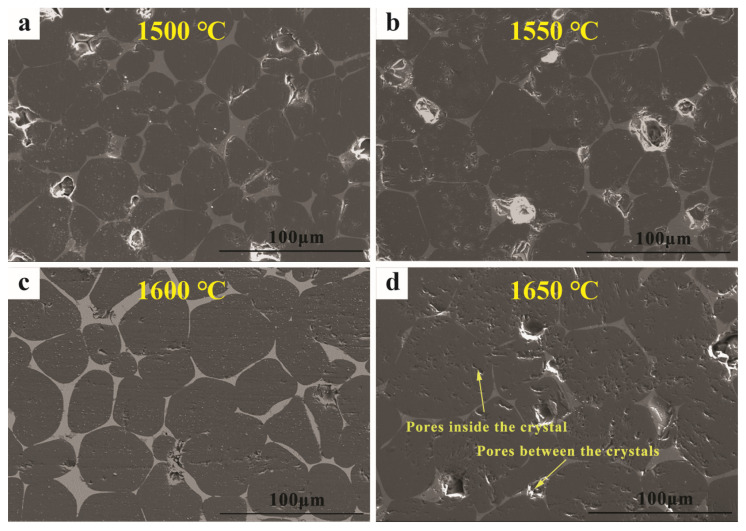
Microstructure of MgO-CaO-Fe_2_O_3_ clinker fired at different temperatures. (**a**) 1500 °C (**b**) 1550 °C (**c**) 1600 °C (**d**) 1650 °C.

The microstructures of the (MCF)cl specimens after firing at different temperatures are shown in Figure 5, where the round or elliptical MgO grains in (MCF)cl are uniformly distributed. When comparing the size of the MgO grains in the fired specimens, it can be observed that the size of the MgO grains is smallest after firing at 1500 °C (15–50 μm), comparable after firing at 1550 °C and 1600 °C (20–60 μm), and largest for the specimen fired at 1650 °C (30–80 μm). Therefore, an increase in firing temperature promotes the growth of MgO grains. However, the distribution of pores in the specimens after firing at different temperatures was not the same, and the intercrystalline and intergranular porosity was reduced in the specimens after firing at 1600 °C. The MgO-CaO-FeO_n_-Al_2_O_3_-SiO_2_ multiple systems are known to form the liquid phase at 1250 °C [5]. Thus, the high-temperature sintering process of (MCF)cl is also liquid phase sintering, as attested by the large quantity of liquid phase produced. The liquid phase sintering is driven by surface tension and achieves pore filling and grain growth through flow-transfer and dissolution-precipitation processes [18]. As the firing temperature increases to 1600 °C, the flow rate of mass transfer is accelerated, the grains gradually grow, the pores in the specimen are reduced, and the bulk density of the specimen particles gradually increases. The firing temperature continues to rise to 1650 °C, but due to the rapid growth of the crystal, the rate of movement of the grain boundary is greater than the rate of movement of the pores, making it too late to discharge the pores. At the same time, due to the growth of MgO grains and the formation of local direct bonding, the liquid phase between the grains will be directly expelled, forming intergranular pores, as shown in Figure 5d. As a result, compared to the specimens fired at 1600 °C, the water absorption of the specimens fired at 1650 °C increased.

Table 2 demonstrates the bulk density of (MCF)cl fired at 1600 °C. The heating-permanent line variation and compressive strength of the crushed and reformed specimens refired at different temperatures, and the requirements of the standard (YB/T 101-2018) for the DHL-81 grade product, are also shown in Table 2. As can be seen, the bulk density of (MCF)cl exceeded the requirements of the standard, and the heating-permanent line variation and compressive strength of the specimens after re-firing at 1300 °C and 1600 °C met the requirements of the standard, respectively.

### 3.2. Synthesis Mechanism of MgO-CaO-Fe_2_O_3_ Clinker

**Figure 6:**
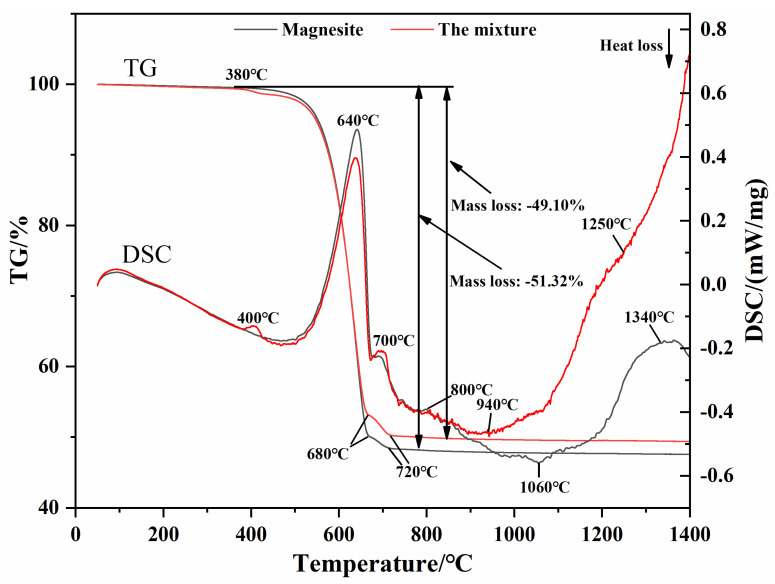
Thermal analysis curves of the magnesite and the mixture.

Figure 6 shows the TG-DSC thermal analysis curves of the magnesite used for the preparation of (MCF)cl and the mixture of different raw materials. As can be seen, magnesite and the mixture have similar DSC curve shapes, both having two heat-absorption peaks at about 640 °C and 700 °C, corresponding to two weight loss steps on the TG curve, followed by a larger exothermic peak starting at about 800 °C. The difference lies in the fact that the mixture has a lower heat-absorption peak at about 400 °C, corresponding to a small weight loss step on the TG curve. Comparing the peak shapes and peak areas of the DSC curves of the two materials, the peak area of the heat absorption of the mixture at 640 °C is smaller than that of magnesite, i.e., the thermal effect is smaller, while the peak area of the heat absorption at 700 °C is larger than that of magnesite. The peak temperature of the heat-absorption peak of the mixture beginning at 800 °C is low (940 °C), and the exothermic reaction is still happening until 1400 °C, while the peak temperature of magnesite is higher (1060 °C) and the exothermic reaction ends at around 1340 °C.

Since magnesite contains a small amount of dolomite, and the CaO is used after digestion, the main composition of the mixture is MgCO_3_/Ca(OH)_2_/CaMg(CO_3_)_2_/Fe_2_O_3_; the chemical reactions that may occur in this composition system during heating are as follows:

(1) $$Ca{\left(OH\right)}_{2}=CaO+H_{2}O↑$$

(2) $$MgCO_{3}=MgO+CO_{2}↑$$

(3) $$CaMg{\left(CO_{3}\right)}_{2}=CaO+MgO+2CO_{2}↑$$

(4) $$MgCO_{3}+CaO=CaCO_{3}+MgO$$

(5) $$2MgCO_{3}+CaO=CaMg{\left(CO_{3}\right)}_{2}+MgO$$

(6) $$CaCO_{3}=CaO+CO_{2}↑$$

(7) $$2CaO+Fe_{2}O_{3}=2CaO\cdot Fe_{2}O_{3}$$

**Figure 7:**
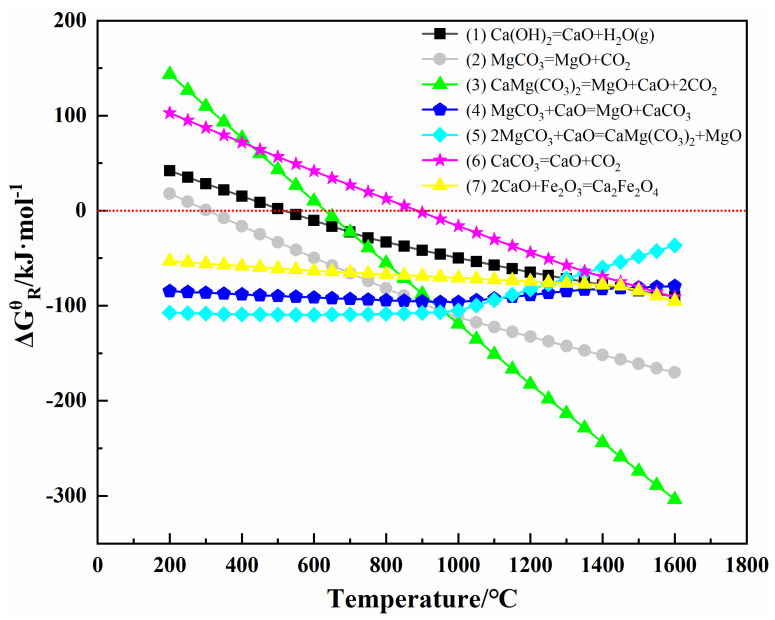
Relationships between Gibbs free energy change and the temperatures of the reactions.

The standard Gibbs free energy change for the above reaction equations (1)–(7) was calculated using the thermodynamic simulation software HSC Chemistry 6; the results are shown in Figure 7. The figure shows that the standard Gibbs free energy change of reaction Equations (1)–(3) gradually decreases with the increasing simulation temperature, and that the standard Gibbs free energy change of the reaction decreases below zero after the temperature rises to a certain level, indicating that these three reactions can gradually proceed spontaneously with increasing temperature. Combining the peak temperature on the DSC curve with the size of the weight loss steps on the TG curve in Figure 6, it can be seen that reaction Equation (1) corresponds to the heat-absorption peak at 400 °C on the DSC curve, reaction Equation (2) corresponds to the heat-absorption peak at 640 °C on the DSC curve, and reaction Equation (3) corresponds to the heat-absorption peak at 700 °C on the DSC curve. Again, as can be seen in Figure 7, the standard Gibbs free energy change for reaction Equations (4), (5), and (7) is always less than zero throughout the simulated temperature interval, indicating that all three reactions can proceed spontaneously. At the same time, the thermodynamic driving force of Reaction (5) is stronger because the standard Gibbs free energy change has the smallest value at the same temperature (<1000 °C). In contrast, the mixture system contains an excess of MgCO_3_, so from the beginning of Ca(OH)_2_ decomposition until the temperature stage before CaMg(CO_3_)_2_ decomposition (380–680 °C), reaction Equation (5) will occur preferentially, i.e., the CaO generated by the decomposition of Reaction (1) will then react with MgCO_3_ to form CaMg(CO_3_)_2_.

Table 3 shows the thermodynamic simulation data for reaction Equations (2), (5), and (7). The results show that in the synthesis of (MCF)cl, Reaction (2) is a heating-absorbing reaction, while Reaction (5) is exothermic, i.e., compared to magnesite without the presence of Ca(OH)_2_, the total value of energy absorbed by the system in the mixture is reduced because of Reaction (5), so the peak area of heat absorption of MgCO_3_ decomposition at 640 °C in the mixture is smaller than that of magnesite. The production of CaMg(CO_3_)_2_ in Reaction (5) also increases the total CaMg(CO_3_)_2_ content of the mixture relative to magnesite. Consequently, the area of the heat-absorption peak formed by the decomposition of CaMg(CO_3_)_2_ at 700 °C in the mixture is larger than that of magnesite.

It is known that the decomposition of CaMg(CO_3_)_2_ can be divided into two stages as follows [19]:

(8) $$nCaMg{\left(CO_{3}\right)}_{2}\to \left(n-1\right)MgO+MgCO_{3}\cdot nCaCO_{3}+\left(n-1\right)CO_{2}↑$$

(9) $$MgCO_{3}\cdot nCaCO_{3}\to MgO+CaO+\left(n+1\right)CO_{2}↑$$

In this case, the decomposition temperature of MgCO_3_ in the first-stage Reaction (8) is 700 °C, and the decomposition temperature of CaCO_3_ in the second-stage Reaction (9) is 900 °C, while no heat-absorption peak for the decomposition of CaCO_3_ can be found in the thermal analysis curve. As the standard Gibbs free energy change for the formation of 2CaO·Fe_2_O_3_ in reaction Equation (7) is also less than zero (see Figure 7), when CaCO_3_ starts to decompose, Reaction (7) also proceeds simultaneously. Reaction (7) is exothermic, and the exothermic growth of periclase grains is taking place at the same time, so there is only one large exothermic peak in the DSC curve of the mixture from 800 °C onward, i.e., a combination of three energy changes: the heat absorption of CaCO_3_ decomposition, the exotherm of 2CaO·Fe_2_O_3_ generation, and the exotherm of periclase grain growth. In comparison with the mixture, the contents of CaO and Fe_2_O_3_ in magnesite are much lower, and the exothermic peak after 800 °C is mainly the exothermic growth of periclase grains. Furthermore, the exothermic effect is not concentrated compared to the mixture, so its peak temperature is higher than the mixture.

While the temperature continues to rise, due to the unique decomposition characteristics of magnesite, decomposition still maintains the original particle form, forming a loose porous structure [20,21], making the growth of MgO grains difficult. Thus, the exothermic reaction of magnesite at 1340 °C finishes, and the grain growth stops. However, the mixture produces a liquid phase when heated to 1250 °C, as evidenced by the turnaround of its DSC curve at 1250 °C. The presence of the liquid phase accelerates the mass transfer rate and promotes the growth of periclase grains. Therefore, the exothermic reaction of the mixture continues up to 1400 °C, i.e., periclase grains continue to grow.

**Figure 8:**
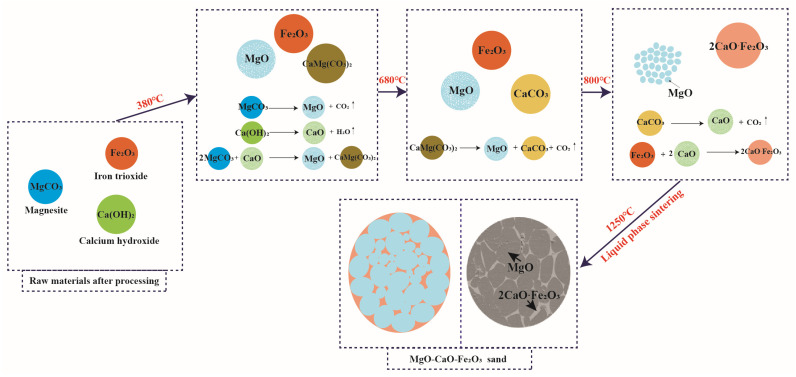
Synthesis mechanism of the MgO-CaO-Fe_2_O_3_ clinker.

In summary, as shown in Figure 8 for the synthesis mechanism of (MCF)cl, when the firing temperature reaches 380–680 °C, MgCO_3_ and Ca(OH)_2_ in the raw material start to decompose to form CaO, which will react with MgCO_3_ to form CaMg(CO_3_)_2_. As the firing temperature rises to 680–720 °C, CaMg(CO_3_)_2_ decomposes to form CaCO_3_. Subsequently, with the firing temperature rising further to 800–1250 °C, CaCO_3_ begins to decompose, while the resulting CaO reacts with Fe_2_O_3_ to form 2CaO·Fe_2_O_3_, and MgO grains gradually grow. Finally, when the firing temperature is greater than 1250 °C, the liquid phase begins to appear in the raw material system, and the liquid phase is distributed in the MgO intergranular grains. In the liquid phase, with the promotion of mass transfer, MgO grains continue to grow, and the specimen begins to densify.

## 4. Conclusions

High-performance (MCF)cl with a bulk density of 3.42 g·cm^−3^, water absorption of 0.7%, heating-permanent line change, and compressive strength greater than the standard (YB/T 101-2018) was prepared using magnesite with high calcium and low silica from Xinjiang, calcium oxide, and ferric oxide. The main crystalline phase of the prepared (MCF)cl is MgO, and a microstructure of 2CaO·Fe_2_O_3_ cemented MgO is formed between the MgO grains, with small quantities of 3CaO·SiO_2_ and 4CaO·Al_2_O_3_·Fe_2_O_3_ also distributed between the MgO grains. During the preparation of (MCF)cl, the synthesis and re-decomposition of CaMg(CO_3_)_2_ occur in the system as the temperature rises, and the liquid phase that appears in the system fills the intergranular spaces of the MgO grains. The mass-transfer methods based on high-temperature liquid phases promote the continuous growth of MgO grains and increase the densification of (MCF)cl.

## Figures and Tables

**Table 1:** Chemical composition of magnesite (wt%).

	MgO	CaO	SiO_2_	Al_2_O_3_	Fe_2_O_3_	LoI
Magnesite	44.97	1.99	0.43	0.13	0.58	51.95

**Table 2:** Physical properties of MgO-CaO-Fe_2_O_3_ clinker fired at 1600 °C.

	Bulk Density before Refiring /g·cm^3^	Refiring Temperature /°C	Heating-Permanent Line Change after Refiring /%	Compressive Strength after Refiring /MPa
MgO-CaO-Fe_2_O_3_	3.42	1300	−0.5	17.9
		1600	−2.5	39.1
DHL-81	3.25	1300	−0.2~−0.5	≥10
		1600	−2.0~−3.0	≥30

**Table 3:** Thermodynamic simulation data of Reactions (2), (5), and (7).

Temperatures /°C	Reaction (2)		Reaction (5)		Reaction (7)	
	ΔH /kJ·mol	ΔG /kJ·mol	ΔH /kJ·mol	ΔG /kJ·mol	ΔH /kJ·mol	ΔG /kJ·mol
200	100.004	17.989	−102.077	−107.386	−40.330	−52.972
300	98.986	0.744	−103.502	−108.377	−40.405	−55.644
400	97.583	−16.291	−105.406	−109.077	−40.907	−58.269
500	95.773	−33.088	−107.764	−109.461	−41.980	−60.781
600	93.530	−49.624	−110.566	−109.511	−43.895	−63.106
700	90.828	−65.877	−113.812	−109.215	−46.860	−65.143
800	87.649	−81.829	−117.507	−108.563	−47.118	−67.001
900	83.970	−97.461	−121.656	−107.545	−47.009	−68.859
1000	20.807	−112.291	−244.197	−105.221	−46.961	−70.724
1100	16.795	−122.592	−247.847	−94.159	−47.025	−72.590
1200	12.892	−132.605	−250.672	−82.859	−47.228	−74.445
1300	9.089	−142.356	−252.678	−71.396	−47.578	−76.282
1400	5.378	−151.868	−253.877	−59.829	−48.092	−78.092
1500	1.754	−161.160	−254.277	−48.215	105.501	−84.387

## Data Availability

The data presented in this study are available on request from the corresponding author.
